# Risk of Erectile Dysfunction Induced by Arsenic Exposure through Well Water Consumption in Taiwan

**DOI:** 10.1289/ehp.10930

**Published:** 2008-01-16

**Authors:** Fang-I Hsieh, Ti-Sheng Hwang, Yi-Chen Hsieh, Hsiu-Chiung Lo, Chien-Tien Su, Hui-Shing Hsu, Hung-Yi Chiou, Chien-Jen Chen

**Affiliations:** 1 School of Public Health and; 2 Topnotch Stroke Research Center, Taipei Medical University, Taipei, Taiwan; 3 Division of Urology, Department of Surgery, Shin Kong Wu Ho-Su Memorial Hospital, Taipei, Taiwan; 4 Department of Family Medicine, Taipei Medical University Hospital, Taipei, Taiwan; 5 Department of Urology, Lotung Poh-Ai Hospital, Lotung, Taiwan; 6 Genomics Research Center, Academia Sinica, Taipei, Taiwan

**Keywords:** arsenic exposure, calculated free testosterone, erectile dysfunction, sex hormone, total testosterone

## Abstract

**Background:**

Erectile dysfunction (ED) has a profound impact on the quality of life of many men. Many risk factors are associated with ED, such as aging, sex hormone levels, hypertension, cardiovascular diseases, and diabetes mellitus. Arsenic exposure could damage peripheral vessels and increase the risk of cardiovascular disease. However, the relationship between arsenic exposure and ED has seldom been evaluated.

**Objectives:**

In this study we aimed to investigate whether exposure to arsenic enhances the risk of ED.

**Methods:**

We recruited 177 males ≥ 50 years of age through health examinations conducted in three hospitals in Taiwan. We used a questionnaire (International Index of Erectile Function-5) to measure the level of erectile function. Sex hormones, including total testosterone and sex hormone–binding globulin, were determined by radioimmunoassay. We used another standardized questionnaire to collect background and behavioral information (e.g., cigarette smoking; alcohol, tea, or coffee drinking; and physical activity).

**Results:**

The prevalence of ED was greater in the arsenic-endemic area (83.3%) than in the non–arsenic-endemic area (66.7%). Subjects with arsenic exposure > 50 ppb had a significantly higher risk of developing ED than those with exposure ≤ 50 ppb, after adjusting for age, cigarette smoking, diabetes mellitus, hypertension, and cardiovascular disease [odds ratio (OR) = 3.4]. Results also showed that the risk of developing severe ED was drastically enhanced by arsenic exposure (OR = 7.5), after adjusting for free testosterone and traditional risk factors of ED.

**Conclusions:**

Results suggested that chronic arsenic exposure has a negative impact on erectile function.

According to the definitions of the First International Consultation on erectile dysfunction (ED), co-sponsored by the World Health Organization (WHO), ED is considered “the consistent or recurrent inability to attain and/or to maintain a penile erection sufficient for sexual performance” (Jardin et al. 2000). Although ED is a benign disorder, it is related to physical and psychological health, and it has a notable impact on the quality of life of sufferers and their families (Wespes et al. 2002).

Aging is the most common risk factor associated with ED. Therefore, age-related factors, including hormonal derangement, diabetes mellitus, neural damage from surgery, side effects of drug, radiation therapy, and psychogenic factors, are the most frequently cited causes of ED (Araujo et al. 1998; Wein and van Arsdalen 1988). Of the sex hormone levels, the changes in free testosterone correlate most closely with aging and have the closest correlation with sexual activity (Ahn et al. 2002). Androgens have been suggested to be essential in the maintenance of libido and important in regulating penile smooth muscle function in men (Salonia et al. 2003). In animal models, androgens can regulate the expression and activity of nitric oxide synthase (NOS) isoforms in the corpus cavernosum (Marin et al. 1999; Park et al. 1999; Shen et al. 2000). Nitric oxide (NO) was considered to mediate relaxation of the vascular smooth muscle of the resistance arteries of the corpus cavernosum and the trabeculae to facilitate penile erection (Andersson and Wagner 1995; Burnett et al. 1992; Moreland et al. 2001). In castrated animals, testosterone and 5α-dihydro-testosterone administration restored the erectile response and NOS expression in the penis (Armagan et al. 2006; Baba et al. 2000a, 2000b). Within the follow-up data of the Massachusetts Male Aging Study, total testosterone declined 0.8%/year (Feldman et al. 2002), whereas both free and albumin-bound testosterone declined about 1.4–2%/year (Feldman et al. 2002; Vermeulen et al. 1991). In our previous study (Hwang et al. 2007), we found a significant association between low levels of serum calculated free testosterone (cFT), bioavailable testosterone, and severity of ED in middle-aged and aged males in Taiwan.

Accumulated evidence supports the association between ED and cardiovascular risk factors such as hypertension and hyperlipidemia, as well as cardiovascular disease (Billups 2005). Arsenic exposure has been reported to damage peripheral vessels and cause black foot disease (Ch’i and Blackwell 1968). Arsenic exposure has also been correlated with cardiovascular disease such as atherosclerosis (Wang CH et al. 2007; Wang YH et al. 2007; Wu et al. 2006). Zarazua et al. (2006) reported that one possible explanation for the correlation between arsenic exposure and cardiovascular disease is that arsenic exposure is associated with the reduction of NOS activity, which is important in mediating relaxation of the vascular smooth muscle. As mentioned above, NO also plays a critical role in penile erection. However, few studies have discussed the relationship between arsenic exposure and ED. Therefore, our specific aim was to evaluate whether arsenic exposure would increase the risk of ED in males after adjusting for conventional risk factors. We used questionnaires from the International Index of Erectile Function (IIEF-5) (Rosen et al. 1997) to measure the level of erectile function. To our knowledge, this is the first study to investigate the association between ED and arsenic exposure in humans.

## Materials and Methods

### Study subjects

A total of 8,102 residents from 18 villages in four townships were interviewed and recruited as the study cohort during 1991–1994. In 1996, 5,146 subjects from the original cohort, who were still alive and for whom complete information was available for contact address and arsenic levels in well water, were sent letters inviting them to participate in a health examination during 1997–1998; 1,318 cohort members participated (Wu et al. 2006). Because of a limited budget for the present study, we sent invitation letters to a random sample of 300 males ≥ 50 years of age from the 1,318 subjects, asking them to attend a health screening in Lotung Poh-Ai Hospital in 2003. From this group, we recruited 66 males ≥ 50 years of age who lived in a confirmed arsenic-endemic area of Lanyang Basin in Taiwan; the details of arsenic exposure for these subjects have been reported previously (Chiou et al. 1997).

The second group, representing a non–arsenic-endemic area, was made up of 111 males ≥ 50 years of age who were recruited through health examinations in Taipei Wan-Fang Hospital and Taipei Medical University Hospital in 2003.

Using a standardized self-completed questionnaire, we collected information on demographic characteristics (age, marital status, occupation, and education), lifestyle factors (cigarette smoking; alcohol, tea, or coffee drinking; and physical activity), and disease record. We used the IIEF-5 to measure the level of erectile function.

Participants who failed to answer the question on ED or took hormone medication (supplementation or deprivation) were excluded from this study. Using IIEF-5 scores, we assigned all study subjects to ED case (*n* = 129) or control (*n* = 48) groups.

This study was approved by the Institutional Review Board for human subjects of Taipei Medical University. Each subject provided written informed consent prior to the study.

### Definition of erectile function

The maximum score on the IIEF-5 is 25. IIEF-5 scores are characterized as follows: > 21, healthy without ED; 12–21, mild ED; 8–11, moderate ED; and ≤ 7, severe ED.

### Hormone measurement

Nonfasting blood samples (8 mL) were drawn by venipuncture from each study subject during health screening in 2003. All blood samples were collected at 0800–1200 hours and then centrifuged at 3,000 rpm. Serum was stored at –20°C until analysis. Total testosterone and sex hormone-binding globulin (SHBG) were determined by radioimmunoassay. cFT was determined by total testosterone, albumin, and SHBG, using the method of the International Society for the Study of the Aging Male (2007). Serum levels of total testosterone > 11 nmol/L and cFT > 0.23 nmol/L were recognized as normal (Morales et al. 2004).

### Arsenic in well water and arsenic exposure

Well water samples, collected during the home interview, were immediately acidified with hydrochloric acid and then stored at –20°C until analysis. Using hydride generation combined with flame atomic absorption spectrometry, as described previously (Chiou et al. 1997, 2001), we determined the arsenic concentration in the samples. Arsenic concentrations in well water ranged from undetectable (< 0.15 ppb) to 3.59 × 10^3^ ppb.

### Statistical analyses

All data were analyzed using SAS software (version 8.1; SAS Institute Inc., Cary, NC, USA), and were considered statistically significant at *p* < 0.05. We assessed statistical significance using the chi-square test for categorical variables and by analysis of variance (ANOVA) for continuous variables. After completing the ANOVA and finding an effect (rejected the null), we used Duncan’s post hoc test to determine which groups were significantly different from each other. Multivariable logistic regression analyses were employed to estimate odds ratios (ORs) and 95% confidence intervals (CIs). We used models I–III to study the relationships between ED and arsenic exposure, testosterone, and free testosterone, respectively, adjusting for age, smoking, diabetes mellitus, hypertension, and cardiovascular disease. Model IV was used to investigate the relationship between ED and arsenic exposure, adjusting for age, free testosterone, smoking, diabetes mellitus, hypertension, and cardiovascular disease. The same methodology was applied to explore the relationship between severe ED and arsenic exposure.

## Results

The characteristics of study subjects from arsenic-endemic and non–arsenic-endemic areas are shown in Table 1. We found no significant difference in the distributions of age, hypertension, diabetes mellitus, and cardiovascular disease between study subjects from these two areas. The average ages were 67.8 and 67.4 years in the arsenic-endemic and non–arsenic-endemic areas, respectively. The percentage of cigarette smoking was significantly higher in subjects from the arsenic-endemic area than in those from the non–arsenic-endemic area. Interestingly, the prevalence of ED was greater in the arsenic-endemic area (83.3%) than in the non–arsenic-endemic area (66.7%).

The safe level for arsenic in drinking water of 10 ppb, set by the U.S. Environmental Protection Agency (2007), was based on estimation of cancer risk. However, Wang YH et al. (2007) observed a marked age- and sex-adjusted OR of 3.3 for the development of carotid atherosclerosis among subjects in a high-arsenic exposure group who drank well water containing > 50 ppb arsenic; the risk of carotid atherosclerosis was not significant among the group with arsenic exposures of 10–50 ppb. Therefore, we classified subjects into two groups: arsenic exposure > 50 ppb and ≤ 50 pp. Each group was further divided into three subgroups according to IIEF-5 scores (i.e., > 21, 8–21, and < 8) in order to investigate the relationship between sex hormone, arsenic exposure, and ED. As shown in Table 2, significantly lower levels of testosterone or free testosterone were observed in severe ED cases compared with healthy controls. Moreover, both arsenic exposure groups (≤ 50 ppb and > 50 ppb) showed marked linear trends of free testosterone across different severities of ED. However, in the case of testosterone, this occurred only in the group with arsenic exposure ≤ 50 ppb. Without considering ED, we found lower average levels of testosterone and free testosterone in subjects with arsenic exposure > 50 ppb. The average level of SHBG was not significantly different between subjects with or without arsenic exposure. We observed a significant linear trend of SHBG across different severities of ED only in subjects with arsenic exposure > 50 ppb.

To further clarify the relationships between ED and testosterone, free testosterone, and arsenic exposure, we adjusted for traditional risk factors (e.g., age, smoking, diabetes mellitus, hypertension, cardiovascular disease) in models I–III (Table 3). Only subjects with arsenic exposure > 50 ppb (model I) or free testosterone < 0.23 nmol/L (model III) possessed significant risk of ED. For arsenic exposure > 50 ppb in model I, OR = 3.4 (95% CI, 1.1–10.3); for free testosterone < 0.23 nmol/L in model III, OR = 4.8 (95% CI, 1.3–18.0). We found no significant risk of ED among subjects with testosterone < 11 nmol/L in model II. Because free testosterone < 0.23 nmol/L was an important risk factor of ED in model III, we adjusted for free testosterone in model IV to derive a more apparent relationship between arsenic exposure and ED. After adjusting for serum free testosterone and traditional risk factors of ED, arsenic exposure still enhanced the risk of developing ED (OR = 3.0; 95% CI, 1.0–9.2) in model IV. We observed a significant positive trend between age and ED in all models. Moreover, study subjects > 70 years of age had the highest risk of developing ED (OR = 6.0–7.0). As shown in Table 4, we used the same methodology to obtain the ORs of severe ED among subjects with arsenic exposure > 50 ppb. In model IV (Table 4), we observed a drastically enhanced OR (7.5; 95% CI, 1.8–30.9) for severe ED in subjects with arsenic exposure > 50 ppb after adjusting for age, free testosterone, diabetes mellitus, and cardiovascular disease. Other than arsenic exposure, the risk factors for severe ED in model IV included age > 70 years (OR = 30.9; 95% CI, 5.2–182.1), abnormal serum free testosterone level (OR = 4.7; 95% CI, 1.2–18.9), diabetes mellitus (OR = 5.5; 95% CI, 1.2–24.2), and cardiovascular disease (OR = 1.0; 95% CI, 1.0–26.8). We used the current WHO drinking water guideline of 10 ppb (WHO 2006) as a cut point to analyze the data again. The OR of abnormal erectile function among subjects with arsenic exposure > 10 ppb was 1.9 (95% CI, 0.7–5.3), which is not statistically significant after adjusting for age, free testosterone, smoking, diabetes mellitus, hypertension, and cardiovascular disease (data not shown). This indicates that there was no significant risk of abnormal erectile function among subjects with arsenic exposure > 10 ppb. However, for severe ED we observed a significant OR of 4.0 (95% CI, 1.2–13.6) in subjects with arsenic exposure > 10 ppb after adjusting for age, free testosterone, smoking, diabetes mellitus, hypertension, and cardiovascular disease (data not shown). The results showed that the cut point of 50 ppb for arsenic exposure revealed a stronger effect of arsenic in ED than when 10 ppb was used as a cut point.

## Discussion

In the present study, the prevalence of ED in arsenic-endemic and non–arsenic-endemic areas was 83.3% and 66.7%, respectively (Table 1). Ansong et al. (2000) used a self-administered survey to study 5,198 randomly selected men 50–76 years of age living in four rural counties in central New York State. They found age-specific prevalence of ED of 26.0%, 34.9%, 46.9%, 57.8%, and 69.4% among men 50–54, 55–59, 60–64, 65–69, and 70–76 years of age, respectively. In a population-based sample of 50- to 75-year-old Finnish men, Shiri et al. (2003) estimated the overall prevalence of ED to be 76.5%. The prevalence of ED increased from 67% for men 50 years of age to 89% for those 75 years of age. The studies noted above indicated that ED is a highly prevalent disorder among men > 50 years of age, and the prevalence of ED increased with advancing age. In the present study, age distribution (mean ± SD) was not significantly different between subjects from the arsenic-endemic area (67.77 ± 6.76) and the non–arsenic-endemic area (67.42 ± 8.70), but we observed a greater prevalence of ED in the arsenic-endemic area, suggesting that arsenic may play a role of developing ED.

We observed a lower prevalence of diabetes mellitus and hypertension in study subjects from the arsenic-endemic area than in those from the non–arsenic-endemic area (Table 1), but this was not statistically significant. Because the age distribution was similar in subjects from these two areas, the difference in prevalence of diabetes mellitus and hypertension between these two areas may have been caused by health-examination bias. However, even with a health-examination bias, the prevalence of ED was drastically higher in study subjects from the arsenic-endemic area compared with those from the non–arsenic-endemic area. This indicates a limited influence of this potential bias regarding the observation of a high prevalence of ED in the arsenic-endemic area.

We observed a significant association between serum testosterone concentration, free testosterone concentration, and the severity of ED in the group with arsenic exposure < 50 ppb (Table 2). Interestingly, the average levels of free testosterone and testosterone were significantly lower in the subjects with arsenic exposure > 50 ppb compared with the < 50-ppb arsenic group. The data suggest that arsenic was strongly associated with a lower proportion of free fraction, bioavailable testosterone or total testosterone in circulating blood. Previous studies (Kim 1999; Martínez-Jabaloyas et al. 2006) have supported the theory that sex hormones, especially free testosterone, is associated with sexual function. Therefore, arsenic probably increases the risk of ED through reducing the level of free testosterone. Data from a rat model indicated that arsenic has a suppressive influence on spermatogenesis and on gonadotropin and testosterone release (Sarkar et al. 2003). However, the actual mechanism(s) by which arsenic impairs male reproductive function remains unclear.

Age, diabetes mellitus, cigarette smoking, hypertension, sex hormone, and cardiovascular disease have been reported to be associated with ED. In the present study, two risk factors of ED, diabetes mellitus and cardiovascular disease, reached statistical significance only in the subjects with severe ED. After adjustment for age, free testosterone, diabetes mellitus, cigarette smoking, hypertension, and cardiovascular disease, the increased risk of ED was still found in the subjects with arsenic exposure > 50 ppb (Tables 3 and 4). Therefore, arsenic may also influence erectile function via non–hormone-dependent pathway. Oxidative stress has been suggested to be a major cause of male reproductive failure. Indeed, some studies suggested that toxicity resulting from chronic arsenic exposure was caused by oxidative stress (Liu et al. 2001; Maiti and Chatterjee 2001; Ramanathan et al. 2002, 2003; Santra et al. 2000). Alterations in neural and impaired penile vascular systems were believed to be mainly responsible for ED rates that approached 75% in some reports (Benet and Melman 1995; Hakim and Goldstein 1996). NO, derived from vascular endothelial and neural sources, played a critical role in the early steps of the normal cascade of the penile vasculature and the relaxation of cavernous smooth muscle (Andersson and Wagner 1995; Burnett et al. 1992; Moreland et al. 2001). The presence of oxygen free radicals inactivated NO and reduced its physiologic impact. Direct inactivation of NO, largely by superoxide anions, may be involved in producing impaired cavernosal relaxation (Katusic 1996).

## Conclusion

The present study suggests that chronic arsenic exposure has a negative impact on erectile function. The potential pathways of arsenic exposure leading to ED include the inhibition of the sex hormone level, or reduction of NOS activity to impair the functions of penile smooth muscle and blood vessels. Future work, especially with a larger sample, could further elucidate the interaction of arsenic exposure–, sex hormone–, and oxidative stress–related factors on the risk of ED.

## Figures and Tables

**Table 1:** Characteristics [no. (%)] of study subjects from arsenic-endemic and non–arsenic-endemic areas.

	Arsenic-endemic area^a^	Non–arsenic-endemic area	*p*-Value^b^
Age (years)			
< 60	9 (13.6)	22 (19.8)	0.295
≥ 60	57 (86.4)	89 (80.2)	
Cigarette smoking	47 (72.3)	55 (49.6)	0.003
Diabetes mellitus	4 (6.1)	17 (15.3)	0.066
Hypertension	15 (22.7)	38 (34.2)	0.106
Cardiovascular disease	6 (13.6)	16 (14.4)	0.900
ED	55 (83.3)	74 (66.7)	0.016

a The average duration of arsenic exposure was 42 years.

b Determined by chi-square test.

**Table 2:** Sex hormone levels (mean ± SD) of study subjects categorized by erectile function and arsenic exposure.

		ED status					
	Arsenic exposure (ppb)	Normal (*n* = 48)	Moderate/mild (*n* = 49)	Severe (*n* = 77)	*p*-Value for trend	Total (*n* = 174)	*p*-Value^a^
Testosterone (nmol/L)	≤ 50^b^	19.28 ± 7.21^c^	17.50 ± 4.28^cd^	16.03 ± 5.28^d^	0.01	17.55 ± 5.85	
	> 50	16.74 ± 4.19	15.57 ± 2.96	14.46 ± 5.16	0.22	15.04 ± 4.64	0.009
Free testosterone (nmol/L)	≤ 50^b^	0.42 ± 0.17^c^	0.40 ± 0.13^cd^	0.33 ± 0.17^d^	0.01	0.38 ± 0.16	
	> 50	0.39 ± 0.11	0.34 ± 0.09	0.28 ± 0.14	0.03	0.31 ± 0.13	0.003
SHBG (nmol/L)	≤ 50	33.53 ± 15.74	31.25 ± 14.21	37.31 ± 17.03	0.26	34.20 ± 15.86	
	> 50	28.71 ± 11.57	31.29 ± 11.34	41.79 ± 19.17	0.03	37.61 ± 17.50	0.23

ED status: normal, IIEF > 21; moderate/mild, IIEF = 8–21; severe, IIEF ≤ 7.

a Compared with ≤ 50 ppb arsenic exposure.

b Duncan’s post hoc test was used to determine which groups were significantly different from each other after completing an ANOVA in which an effect (rejected the null) was found.

c,d Different letters indicate significant difference; that is, *^c^* vs. *^d^* represents significant difference, but *^c^* vs. *^cd^* or *^cd^* vs. *^d^* represent no significant difference).

**Table 3:** Multivariate-adjusted ORs and 95% CIs in subjects with ED (IIEF ≤ 21) compared with those with normal erectile function.

	Model I OR (95% CI)	Model II OR (95% CI)	Model III OR (95% CI)	Model IV OR (95% CI)
Age (years)				
< 60	1.0	1.0	1.0	1.0
60–70	1.7 (0.6–4.7)	1.9 (0.7–5.0)	1.7 (0.6–4.6)	1.6 (0.6–4.4)
≥ 70	7.0 (2.4–20.6)#	7.0 (2.4–20.7)#	6.0 (2.0–18.1)#	6.1 (2.0–18.4)#
	*p* for trend: < 0.001	*p* for trend: < 0.001	*p* for trend: < 0.001	*p* for trend: < 0.001
Testosterone < 11 (nmol/L)		1.5 (0.5–4.5)		
Free testosterone < 0.23 (nmol/L)			4.8 (1.3–18.0)**	4.3 (1.1–16.7)**
Arsenic exposure (ppb)				
≤ 50	1.0			1.0
> 50	3.4 (1.1–10.3)**			3.0 (1.0–9.2)*
Cigarette smoking	1.2 (0.5–2.5)	1.3 (0.6–2.8)	1.3 (0.6–2.9)	1.2 (0.6–2.7)
Diabetes mellitus	3.0 (0.9–10.6)	2.5 (0.7–8.5)	2.7 (0.8–9.3)	3.2 (0.9–11.0)
Hypertension	0.5 (0.2–1.3)	0.4 (0.2–1.1)	0.4 (0.2–1.0)	0.5 (0.2–1.2)
Cardiovascular disease	3.6 (0.9–14.0)	3.1 (0.8–11.6)	3.1 (0.8–12.2)	3.4 (0.8–13.4)

Models show relationship between ED and arsenic exposure (model I), ED and testosterone (model II), ED and free testosterone (model III), and ED and arsenic exposure, adjusting for free testosterone (model IV).

* *p* = 0.05.

** *p* < 0.05.

# *p* < 0.005.

**Table 4:** Multivariate-adjusted ORs and 95% CIs in subjects with severe ED (IIEF ≤ 7) compared with those with normal erectile function.

	Model I OR (95% CI)	Model II OR (95% CI)	Model III OR (95% CI)	Model IV OR (95% CI)
Age (years)				
< 60	1.0	1.0	1.0	1.0
60–70	4.9 (1.0–24.0)*	4.5 (1.0–20.6)*	4.3 (0.9–20.7)	4.8 (0.9–25.4)
≥ 70	35.9 (6.5–197.5)#	27.1 (5.4–134.8)#	22.1 (4.3–114.4)#	30.9 (5.2–182.1)#
	*p* for trend: < 0.001	*p* for trend: < 0.001	*p* for trend: < 0.001	*p* for trend: < 0.001
Testosterone < 11 (nmol/L)		2.4 (0.7–7.8)		
Free testosterone < 0.23 (nmol/L)			4.9 (1.3–18.9)**	4.7 (1.2–18.9)**
Arsenic exposure (ppb)				
≤ 50	1.0			1.0
> 50	7.7 (2.0–30.0)#			7.5 (1.8–30.9)**
Cigarette smoking	1.2 (0.4–3.0)	1.4 (0.6–3.7)	1.4 (0.5–3.5)	1.2 (0.4–3.2)
Diabetes mellitus	5.9 (1.3–27.22)**	3.8 (1.0–15.2)	3.7 (0.9–14.5)	5.5 (1.2–24.2)**
Hypertension	0.5 (0.2–1.5)	0.4 (0.1–1.1)	0.4 (0.1–1.2)	0.4 (0.1–1.4)
Cardiovascular disease	6.5 (1.3–32.1)**	4.4 (1.0–19.4)*	4.2 (0.9–19.3)	5.3 (1.0–26.8)**

Models show relationship between severe ED and arsenic exposure (model I), severe ED and testosterone (model II), severe ED and free testosterone (model III), and severe ED and arsenic exposure, adjusting for free testosterone (model IV).

* *p* = 0.05.

** *p* < 0.05.

# *p* < 0.005.
